# TGF-β Prodomain Alignments Reveal Unexpected Cysteine Conservation Consistent with Phylogenetic Predictions of Cross-Subfamily Heterodimerization

**DOI:** 10.1534/genetics.119.302255

**Published:** 2019-12-16

**Authors:** Robert G. Wisotzkey, Stuart J. Newfeld

**Affiliations:** * Sema4 Genomics, Stamford, Connecticut 06902; † School of Life Sciences, Arizona State University, Tempe, Arizona 85287-4501

**Keywords:** Activin, alignments/trees, arm/bowtie/straitjacket, BMP, cleavage site, heterodimer

## Abstract

Evolutionary relationships between prodomains in the TGF-β family have gone unanalyzed due to a perceived lack of conservation. We developed a novel approach, identified these relationships, and suggest hypotheses for new regulatory mechanisms in TGF-β signaling. First, a quantitative analysis placed each family member from flies, mice, and nematodes into the Activin, BMP, or TGF-β subfamily. Second, we defined the prodomain and ligand via the consensus cleavage site. Third, we generated alignments and trees from the prodomain, ligand, and full-length sequences independently for each subfamily. Prodomain alignments revealed that six structural features of 17 are well conserved: three in the straitjacket and three in the arm. Alignments also revealed unexpected cysteine conservation in the “LTBP-Association region” upstream of the straitjacket and in β8 of the bowtie in 14 proteins from all three subfamilies. In prodomain trees, eight clusters across all three subfamilies were present that were not seen in the ligand or full-length trees, suggesting prodomain-mediated cross-subfamily heterodimerization. Consistency between cysteine conservation and prodomain clustering provides support for heterodimerization predictions. Overall, our analysis suggests that cross-subfamily interactions are more common than currently appreciated and our predictions generate numerous testable hypotheses about TGF-β function and evolution.

SECRETED TGF-β family members perform a myriad of tasks during development and homeostasis, while mutations disrupting TGF-β pathways can lead to disease. The mouse genome encodes 33 TGF-β family members, the fly encodes seven, and the nematode encodes five (Kahlem and Newfeld 2009). Structurally, TGF-β family members share an amino-terminal signal sequence, a long prodomain involved in regulation that is cleaved before secretion but remains associated, and a short biologically active ligand that binds to cell surface receptors. The ligand of TGF-β proteins contains a stereotypical pattern of six cysteines, with a subset containing seven or nine cysteines that form a disulfide bond–based cystine knot structure [reviewed in Hinck *et al.* (2016)].

One means of generating hypotheses for multigene families is to ascertain evolutionary relationships via phylogenetics. This approach has successfully predicted new mechanisms of TGF-β regulation twice. First, Smad linker phosphorylation was predicted (Newfeld and Wisotzkey 2006) then validated by experiment in mice and flies (Fuentealba *et al.* 2007; Quijano *et al.* 2011). Second, monoubiquitylation of Smad4 was predicted (Konikoff *et al.* 2008) then validated by experiment in frogs, mice, and flies (Dupont *et al.* 2009; Morsut *et al.* 2010; Stinchfield *et al.* 2012).

Twenty years ago the first phylogenetic study of TGF-β ligands employed fly, mouse, and nematode proteins, before all three genomes were available (Newfeld *et al.* 1999). To date, all ligand phylogenetic studies have been done with an artificially shortened ligand that begins at the first conserved cysteine (the cystine knot). Historically this was necessary because the cleavage sites that separate the prodomain from the ligand were not defined. This biased the analysis toward the most highly conserved region. Nevertheless, the resultant clustering of the TGF-β superfamily into two large subfamilies (BMP and Activin + TGF-β) that functionally appeared to rely on distinct sets of receptors and receptor-associated Smads was intellectually satisfying.

The first full-length tree of TGF-β family members utilizing the same species was published 10 years ago, after all three genomes were available (Kahlem and Newfeld 2009). Discrepancies with the previous cystine knot tree were noted. The prodomain sequences responsible for full-length *vs.* cystine knot tree discrepancies have not been identified.

While the prodomain has long been known to be required for proper folding and dimerization of the ligand (Gentry and Nash 1990; Gray and Mason 1990), formal definition of cleavage sites came later (Degnin *et al.* 2004; Kunnapuu *et al.* 2009). Proprotein/latent complex crystal structures of TGF-β1 (Shi *et al.* 2011), BMP9 (Mi *et al.* 2015), and Inhibin-βa (also called Activin-A; Wang *et al.* 2016) and the solution structure of Myostatin (also called GDF8; Walker *et al.* 2018) have identified functional features such as the Latency Lasso and the bowtie.

We hypothesized that the discrepancy between the full-length and the cystine knot trees was due to conserved prodomain sequences involved in dimerization. However, the perception was that prodomains were to degenerate to be confidently aligned. To test our hypothesis, we developed a new approach that began with a quantitative analysis of each family member from fly, mouse, and nematode that sorted them into one of three subfamilies: Activin, BMP, and TGF-β. Then we employed the biochemically defined consensus cleavage site to separate each full-length protein into ligand and prodomain. We generated annotated alignments to examine structural conservation. Lastly, trees of the prodomain, biochemically defined ligand and full-length sequences were created from each individual subfamily, an Activin + TGF-β subfamily and from all family member alignments.

The implementation of the consensus cleavage site led to the movement of a highly degenerate region between the cleavage site and the first cysteine out of the prodomain and into the ligand. This resulted in a reduction in the resolution of our ligand trees (*vs.* cystine knot trees), but an increase in resolution of prodomain trees. In our view, cystine knot clustering suggests common receptor binding and common function, while prodomain clustering suggests heterodimerization and common regulation.

In the interest of brevity, we focus our analysis on fly proteins plus interesting observations for nematode family members and mouse Nodal. The prodomain alignments revealed that six structural features are well conserved: three in the straitjacket and three in the arm. Alignments also revealed unexpected cysteine conservation in the “Latent TGF-β Binding (LTBP) LTBP association region” upstream of the straitjacket and in β8 of the arm in 14 proteins belonging to all three subfamilies. In the prodomain trees, eight clusters across all three subfamilies were present that were not seen in the ligand or full-length trees, suggesting prodomain-mediated cross-subfamily heterodimerization. Consistency between cysteine conservation and prodomain clustering provides support for our heterodimerization predictions.

## Materials and Methods

### Sequences and subfamilies

For consistency with our previous papers we focus on the same three species (Newfeld *et al.* 1999; Kahlem and Newfeld 2009). The justification for this approach is that examining genetic model organisms with completely sequenced genomes and an established evolutionary divergence of over a billion years will provide metazoan scale explanatory power and a convenient platform for testing new hypotheses. The newest version of the longest isoform of each TGF-β protein from *Caenorhabditis elegans* (Ce, 5), *Drosophila melanogaster* (Dm, 7), and *Mus musculus* (Mm, 33) was identified. Two species are coelomates with three germ layers and a digestive tract with two openings: *M. musculus* is a deuterostome (blastopore becomes the anus) and *D. melanogaster* is a protostome (blastopore becomes the mouth). *C. elegans* is a pseudocoelomate with three germ layers and a digestive tract with one opening. The split between deuterostomes and protostomes was roughly 964 MYA and between coelomates and pseudocoelomates 1.298 billion years ago (Hedges *et al.* 2004). For consistency with previous papers, mouse GDNF was employed as an outgroup to root all trees. This is appropriate because GDNF shares pattern of cysteines with TGF-β family ligands yet signals strictly via a distinctive ternary complex with Ret tyrosine kinase receptors (*e.g.*, Jing *et al.* 1996). In contrast, Maverick primarily signals through TGF-β receptors but can also bind Ret (Myers *et al.* 2018). This clear distinction in affinity supports our interpretation of data for Maverick. Details on the 46 sequences are in Supplemental Material, Table S1.

Initial separation of fly and mouse TGF-β family members into the two well-known subfamilies Activin/TGF-β and BMP followed Newfeld *et al.* (1999). We then conducted an Informative Sites analyses in MegaX (Kumar *et al.* 2018) to rigorously separate sequences into distinct Activin and TGF-β subfamilies. This had not been done before and led to several changes from previous analyses (Kahlem and Newfeld 2009; Özüak *et al.* 2014). Alignments were generated for the Activin, BMP, and TGF-β subfamilies independently, an Activin + TGF-β combined subfamily, and all family members (five family/subfamilies total).

Separation of full-length sequences into two structural families (prodomain and ligand) was based on identifying the site analogous to the consensus Furin cleavage site in Dpp (Kunnapuu *et al.* 2009; RNKR). For proteins where the sequence was not an exact match and/or there was more than choice, we picked the site closest to the first cysteine in the ligand (Table 2). This approach is more rigorous than past analyses when ligands were defined for convenience at the first conserved cysteine (Newfeld *et al.* 1999). The spacer between the most proximal Furin site and first cysteine in Dpp is 14 residues (Table S3). To validate our cleavage site we checked for conservation in three pairs of congeneric species: *D. melanogaster* and *D. simulans*, *C. elegans* and *C. briggsae*, and *M. musculus* and *M. caroli*. We identified and aligned the region surrounding our chosen cleavage site via BLASTp. The analysis showed that that all fly and mouse cleavage sites are identical in both species, while nematodes showed minor differences in the site in three proteins.

Prodomain, ligand, and full-length trees were analyzed according to subfamily in the main paper. Trees were grouped according to structure (prodomain, ligand, and full-length) in Figures S1–S3. A cystine knot tree for all family members, where the ligand begins at the first cysteine, is included for comparison to the cleavage site defined ligand tree in Figure S2.

### Alignments

Sequences from NCBI were aligned with default settings in Clustal Omega at EMBL-EBI (https://www.ebi.ac.uk/Tools/msa/clustalo/). Alignments depicting sequence conservation were generated in BoxShade3.21 (ch.embnet.org/software/BOX_form.html) as described (Newfeld and Wisotzkey 2006). The cutoff for shading was an identical or similar amino acid in half of the sequences. Similar amino acids are: D/E, K/R/H, N/Q, S/T, I/L/V, F/W/Y, and A/G (Smith and Smith 1990). A set of complete BoxShade alignments for the prodomain, with ungainly leaders and tails trimmed, are found in Figures S4–S8. Fully unedited prodomain as well as ligand and full-length BoxShade alignments are available upon request.

#### Activin subfamily:

We analyzed 11 sequences (1 Ce, 2 Dm, and 8 Mm) plus mouse GDNF. The prodomain alignment was 983 amino acids including gaps, and there were 185 informative sites without gaps. The ligand alignment was 204 amino acids including gaps, and there were 76 informative sites without gaps. The full-length alignment was 1147 amino acids including gaps, and there were 262 informative sites without gaps.

#### TGF-β subfamily:

We analyzed 12 sequences (2 Ce, 2 Dm, and 8 Mm) plus mouse GDNF. The prodomain alignment was 838 amino acids including gaps, and there were 230 informative sites without gaps. The ligand alignment was 167 amino acids including gaps, and there were 101 informative sites without gaps. The full-length alignment was 929 amino acids including gaps, and there were 358 informative sites without gaps.

#### Activin + TGF-β subfamily:

We analyzed 23 sequences (3 Ce, 4 Dm, and 16 Mm) plus mouse GDNF. The prodomain alignment was 1116 amino acids including gaps, and there were 24 informative sites without gaps. The ligand alignment was 214 amino acids including gaps, and there were 76 informative sites without gaps. The full-length alignment was 1302 amino acids including gaps, and there were 101 informative sites without gaps.

#### BMP subfamily:

We analyzed 22 sequences (2 Ce, 3 Dm, and 17 Mm) plus mouse GDNF. The prodomain alignment was 787 amino acids including gaps, and there were 335 informative sites without gaps. The ligand alignment was 166 amino acids including gaps, and there were 108 informative sites without gaps. The full-length alignment was 870 amino acids including gaps, and there were 415 informative sites without gaps.

#### All family members:

We analyzed 45 sequences (5 Ce, 7 Dm, and 33 Mm) plus mouse GDNF. The prodomain alignment was 1265 amino acids including gaps, and there were 554 informative sites without gaps. The ligand alignment was 229 amino acids including gaps, and there were 142 informative sites without gaps. The full-length alignment was 1414 amino acids including gaps, and there were 641 informative sites without gaps. Cystine knot alignment was 168 amino acids including gaps, and there were 114 informative sites without gaps.

### Phylogenetics

Trees were created in MrBayes3.2 (Ronquist *et al.* 2012; mrbayes.sourceforge.net/). The “prior amino acid model” was set to BloSum (a matrix of empirically derived amino acid substitution frequencies; Henikoff and Henikoff 1992) and the “rate of variation across sites” was set to a gamma distribution (this distribution has an L-shape with a few sites evolving rapidly, while most sites are conserved; Yang 1993). Generation times were 200,000 for all trees except that Activin full-length was 100,000. The sample frequency was 100 with burn-in of 0.25.

For alignments with >150 informative positions (prodomain and full-length for all subfamilies except Activin + TGF-β) a posterior probability of 0.95 is statistically significant. For alignments with fewer informative positions, simulation studies (Alfaro *et al.* 2003) showed that the true tree contained branches with posterior probabilities of 0.50 for 25–50 informative positions (Activin + TGF-β prodomain tree), 0.65 for 50–100 informative positions (Activin ligand, TGF-β ligand, and Activin + TGF-β ligand trees), and 0.85 for 100–150 informative positions (BMP ligand, All ligand, cystine knot, and Activin + TGF-β full-length trees).

### Data availability statement

Unedited BoxShade alignments are available upon request. All data necessary for confirming the conclusions are present within the figures, tables, and supplemental information. Supplemental material available at figshare: https://doi.org/10.25386/genetics.11350061.

## Results

### Informative sites analysis and phylogenetics

Given the discordance between previous full-length and cystine knot trees, we began by placing family members rigorously into subfamilies (Table S1). We started with alignments of three sets of recent mammalian duplications that always cluster together and are always distinct from others representing the TGF-β, Activin, and BMP subfamilies (TGF-β1–3; Inhibin-βa, βb, βc, and βe; and BMP2 and 4). Note that the phrase “recent mammalian duplicates” indicates only that these duplications are not present in flies and nematodes.

Then, we added sequences one at a time to each subfamily alignment using the most current version of Clustal Omega (McWilliam *et al.* 2013). Each of these “core plus one” alignments was then run through MegaX (Kumar *et al.* 2018) for a quantitative analysis of total alignment length, gap number, and number of informative sites. A sequence that reduced the number of informative sites by the smallest amount was added to that subfamily and the process repeated until every sequence was added. We did not find any sequences with similar effects on multiple subfamilies as would be expected if there were additional subfamilies.

To our knowledge, this is the first rigorous distinction of sequences within the TGF-β family based on alignment and not a tree-building algorithm. This removes a set of phylogenetic assumptions from the process. Overall, the alignments showed that as a group the TGF-β and Activin subfamilies are just as distinct from each other as they are from BMP, further indicating that there are three separate subfamilies. In the big picture, subfamily separation predates the divergence of flies, mammals, and nematodes, as each subfamily has at least one protein from each species.

The clear distinction between the TGF-β and Activin subfamilies in our sequence analysis is wholly consistent with structural differences between the subfamilies. For example, the TGF-β1 prodomain crystal structure contains a “bowtie” formed by β8 and β9 as part of the closed-ring conformation of the dimer. The bowtie contains cysteines that facilitate dimerization by linking two arm domains together (Shi *et al.* 2011). The bowtie is missing from Inhibin-βa (Wang *et al.* 2016) in the Activin subfamily, whose prodomain structure displays a cross-armed conformation, and from BMP9 (Mi *et al.* 2015), whose prodomain structure exhibits a widely open conformation. Note that BMP9 in this report is present via its synonym GDF2.

Our informative sites analysis led to firm subfamily placement for all proteins in each species. For nematodes, TIG-3 was confirmed in the Activin subfamily; UNC-129 and DAF-7 in the TGF-β subfamily; and TIG-2 and DBL-1 in the BMP subfamily. For flies, the four non-BMP proteins were confidently placed in the Activin (Activin and Myoglianin) and TGF-β subfamilies (Maverick and Dawdle). For mice, Nodal is firmly placed in the BMP subfamily yet our trees will suggest a hypothesis to explain its ability to signal through the Activin pathway receptors ActRIB and ActRIIA/B and signal transducer Smad2 [reviewed Schier (2009)].

Employing a Bayesian approach (Ronquist *et al.* 2012), these rigorous subfamily alignments were built into trees. Confirming our initial hypothesis, these trees were able to resolve conflicts between full-length and cystine knot trees from prior publications. For example, here Gbb and Screw cluster in all trees indicating a recent duplication rather than the complex relationships that were shown previously. In addition, the current approach is better able to discern subtle distinctions between family members. For example, initial placement into subfamilies via informative sites led to 22 BMP proteins that is extended in the current full-length tree of all family members to 26 proteins. This 26-member BMP cluster encompasses two TGF-β and two Activin subfamily members, most likely as a result of previously unsuspected prodomain similarity.

### Cleavage site fidelity and spacer variability

Parsing the full-length sequences into prodomain and ligand, before tree building, based on the consensus Furin cleavage site was not hard (Table S2). Only two of the 46 sequences (45 TGF-β family members plus the mouse GDNF outgroup) did not contain a region with strong similarity to the consensus RX[R/K]R (Degnin *et al.* 2004) upstream of the first cysteine of the ligand. TIG-3 (Activin subfamily) and Maverick (TGF-β subfamily) have only a single R in the right place. In cases where multiple cleavage sites were identified (*e.g.*, Dpp; Kunnapuu *et al.* 2009), we chose the closest R to the first cysteine for the separation. We conducted a similar analysis of known Tolloid cleavage sites that did not reveal any conservation.

To validate our choice of Furin cleavage sites we checked them for conservation in three pairs of congeneric species: *D. melanogaster* and *D. simulans*, *C. elegans* and *C. briggsae*, and *M. musculus* and *M. caroli*. The analysis showed that that all fly and mouse cleavage sites are identical in both species (43 of 46 proteins). Nematodes showed minor differences in three cleavage sites (DAF-7, TIG-2, and TIG-3). An examination of the consensus divergence times between each pair revealed that the fly estimate is 4.7 MYA, the mouse estimate is 4.8 MYA, and the nematode estimate is 60.2 MYA (Timetree.org; Hedges *et al.* 2015). The finding of minor differences in a subset of nematode sequences (three out of five) is unsurprising given the much larger distance between the two species. The high frequency (94%) of identity across species in the cleavage site employed for our analysis provides increased confidence in its validity.

We found that the spacer region between the most proximal cleavage site and the first cysteine was hypervariable in length and content (Table S3). Length variation spanned the range from 2 residues (BMP15; TGF-β subfamily) to 80 residues (BMP3; Activin subfamily). However, in hypervariable regions any conservation likely is functional or evidence of recent duplication. For example, 8 of 11 Activin subfamily members have an acidic residue (D/E) immediately upstream of the first cysteine in the ligand (72% with 10% expected by chance). Only 1 of the other 35 sequences has a glutamic acid at this position. The BMP subfamily has no obvious amino acid conservation but length identity is visible in the recently duplicated Gbb and Screw as well as the mammalian duplicates BMP6, 7, 8a,b. The TGF-β family is home to the two newest duplications as revealed by the presence of both sequence and length identity for Lefty1 and 2 and TGF-β1–3. Overall spacer hypervariability is consistent with structural data showing it sits outside the prodomain-ligand complex (Mi *et al.* 2015).

The transition from a cystine knot defined ligand to a biochemically defined ligand had a dramatic effect on trees for each region. There was a loss of resolution in the ligand tree, as many proteins became unaffiliated. There was a concomitant increase in resolution of the prodomain tree, as a greater number of meaningful clusters are present when compared to the prior full-length tree (Kahlem and Newfeld 2009). Loss of resolution in the ligand tree is of little consequence as a cystine knot alignment of all family members yielded a familiar tree. The gain in resolution for the prodomain revealed numerous unexpected cross-subfamily clusters.

### Trees and alignments of subfamily prodomains, ligands, and full-length proteins

Here data are discussed according to subfamily. For a distinct perspective, the supplemental figures display trees organized by structure (prodomain Figure S1, ligand including cystine knot Figure S2, and full-length Figure S3) and expanded alignments for each subfamily (Figures S4–S8).

### Activin subfamily trees

**Figure 1:**
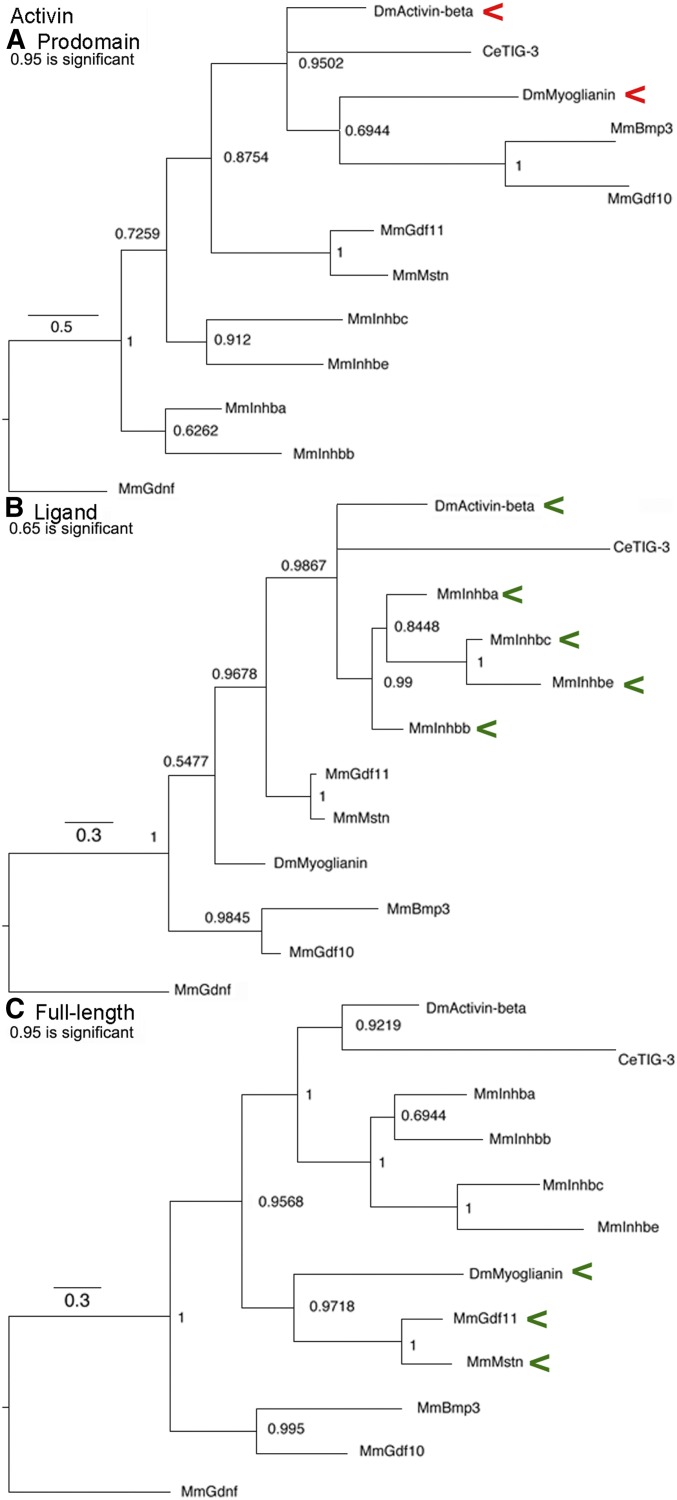
Activin subfamily trees. Bayesian trees of 11 sequences plus the outgroup are displayed. Accession numbers are in Table S1. Branch lengths are drawn to scale and a scale bar indicates the number of amino acid substitutions per site per unit length. Nodes with posterior probabilities ∼0.50 are indicated. Red arrowheads indicate a cluster that may reflect common regulation and green arrowheads a cluster that may reflect common function. (A) Prodomain nodes ∼0.95 are significant. The significant cluster of Activin and Myoglianin is unexpected. (B) Ligand nodes ∼0.65 are significant. The significant cluster of Activin and the four Inhibin-β proteins was expected. (C) Full-length nodes ∼0.95 are significant. The significant cluster of Myoglianin and Myostatin/GDF11 was expected.

This subfamily (Figure 1) is built upon the four Inhibin-β proteins that cluster together in all trees based on their recent origin and common ability to form heterodimers with Inhibin-α in the TGF-β subfamily (Walton *et al.* 2009). The significant cluster of Activin and Myoglianin seen only in the prodomain suggests that they have common regulation. The significant cluster of Activin with the four Inhibin-β proteins in the ligand suggests a common function. The significant cluster of Myoglianin and Myostatin/GDF11 in the full-length tree also suggests common function. Overall for the Activin subfamily, the similarity between the ligand tree and the full-length tree indicates that functional relationships of the ligand are driving its evolution.

### Activin subfamily prodomain structural conservation

**Figure 2:**
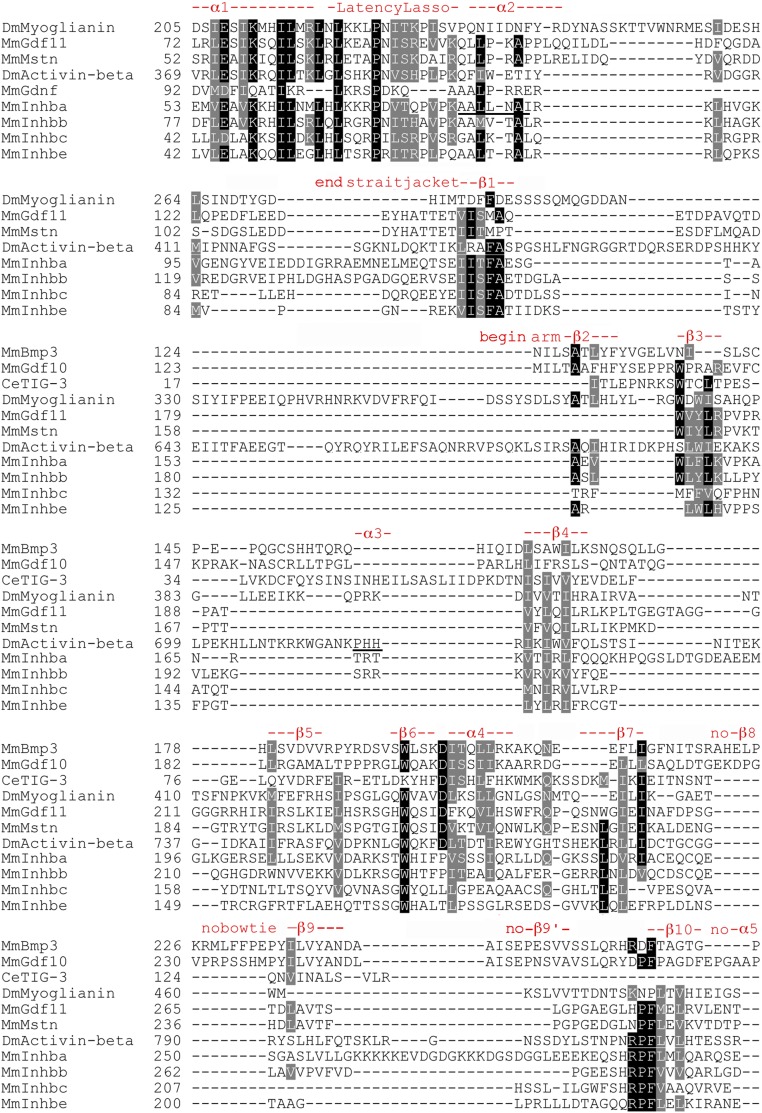
Focused Activin subfamily prodomain alignment indicating structural conservation. Sequences present only, as gaps for any row were omitted. Numbering is accurate. Black shading in indicates an identical and gray shading biochemically similar amino acids at that position. As there is no available structurally annotated full-length sequence of Inhibin-βa, the locations and naming of structural features are derived from our alignment of the Activin + TGF-β subfamily that follows TGF-β1 of Shi *et al.* (2011). Underlined α2 indicates a location distinct in this subfamily from α2 in TGF−β1 and the underlined α3 suggests that this feature of TGF-β1 may be absent in the Activin subfamily. β9′ is not obvious and may be unique to the BMP subfamily.

Known features such as α-helices and β-sheets were located on the alignment revealing pockets of structural conservation in the annotated Activin alignments (Figure 2 and Figure S4). The locations for α1, the Latency Lasso, and α2 are based on Inhibin-βa (Wang *et al.* 2016). The locations of the remaining features derive from our alignment of the Activin + TGF-β subfamily following TGF-β1 (Shi *et al.* 2011).

The four features of the straitjacket domain (α1, the Latency Lasso, α2, and β1) show the most conservation. There is a set of nine I/L/V residues, of which four are universal. There is also a universal proline in the Latency Lasso. β1 contains two conserved I/L/V residues and a phenylalanine. In the arm domain, the helices β2–10 and α4 show less conservation. Most notable are three I/L/V residues in β4, a near universal tryptophan in β6 and near universal I/L/V residues in α4 and β7. This correlation of amino acid conservation with structural features had not been demonstrated rigorously in the Activin subfamily.

### TGF-β subfamily trees

**Figure 3:**
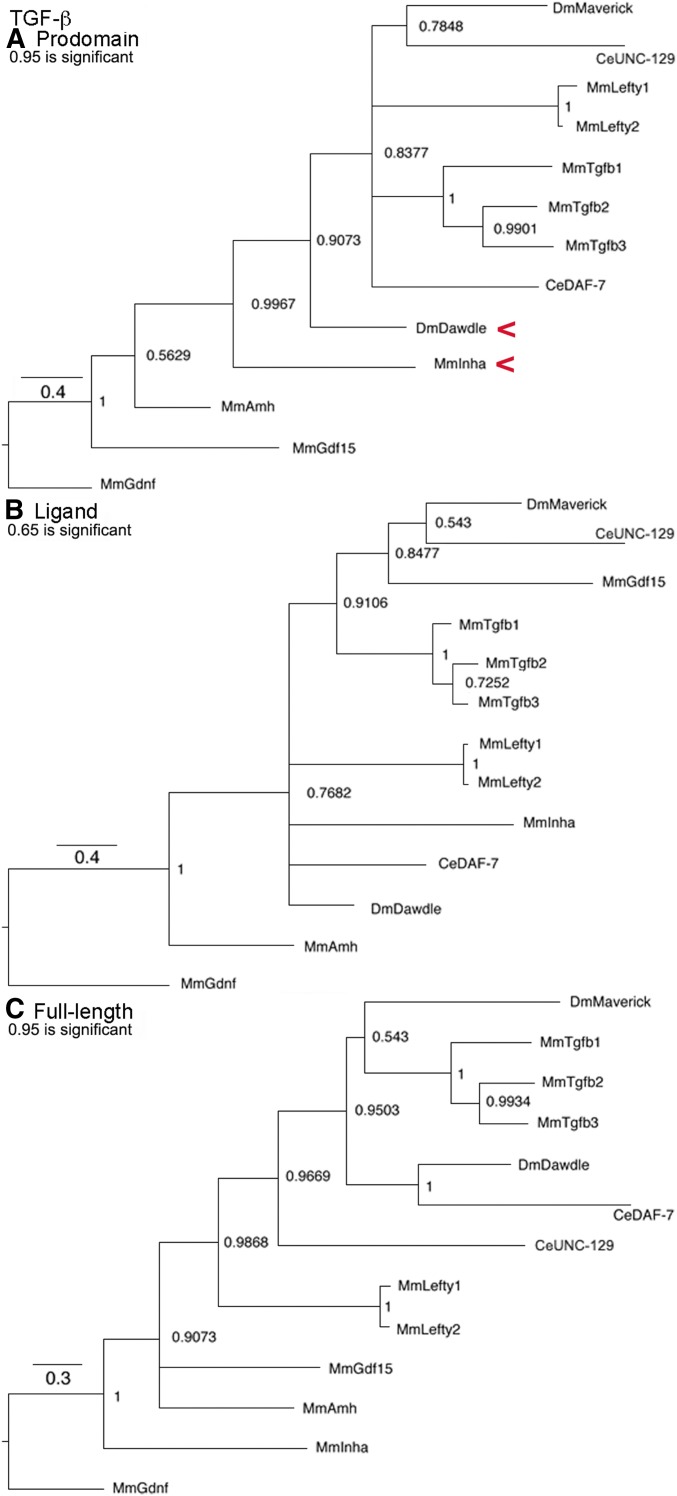
TGF-β subfamily trees. Bayesian trees of 12 sequences plus the outgroup are displayed as in Figure 1. Red arrowheads indicate a cluster that may reflect common regulation. (A) Prodomain nodes ∼0.95 are significant. The significant cluster of Dawdle and Inhibin-α was unexpected. (B) Ligand nodes ∼0.65 are significant. The significant clusters of TGF-β1–3 and Lefty1 and 2 were expected. (C) Full-length nodes ∼0.95 are significant. The significant clusters of TGF-β1–3 and Lefty1 and 2 were expected.

This subfamily (Figure 3) is built upon the three TGF-β proteins that cluster together in all trees, based on their recent origin and common regulation by LTBP (Rifkin *et al.* 2018). Neither prototypical TGF-β nor LTBP are present in flies and neither Maverick nor Dawdle has any relationship with them. The significant cluster of Dawdle and Inhibin-α seen only in the prodomain suggests common regulation. Given the ability of Inhibin-α to form heterodimers and the previously noted cluster of Activin and the Inhibin-β group, Dawdle is a candidate as a heterodimerization partner with Activin. The ligand tree shows established clusters such as TGF-β1–3 and Lefty1, 2 and new clusters such as Maverick with GDF15. Overall for the TGF-β subfamily, the full-length tree is distinct from the ligand and prodomain trees, indicating that functional and regulatory relationships are equally driving its evolution.

### TGF-β subfamily prodomain structural conservation

**Figure 4:**
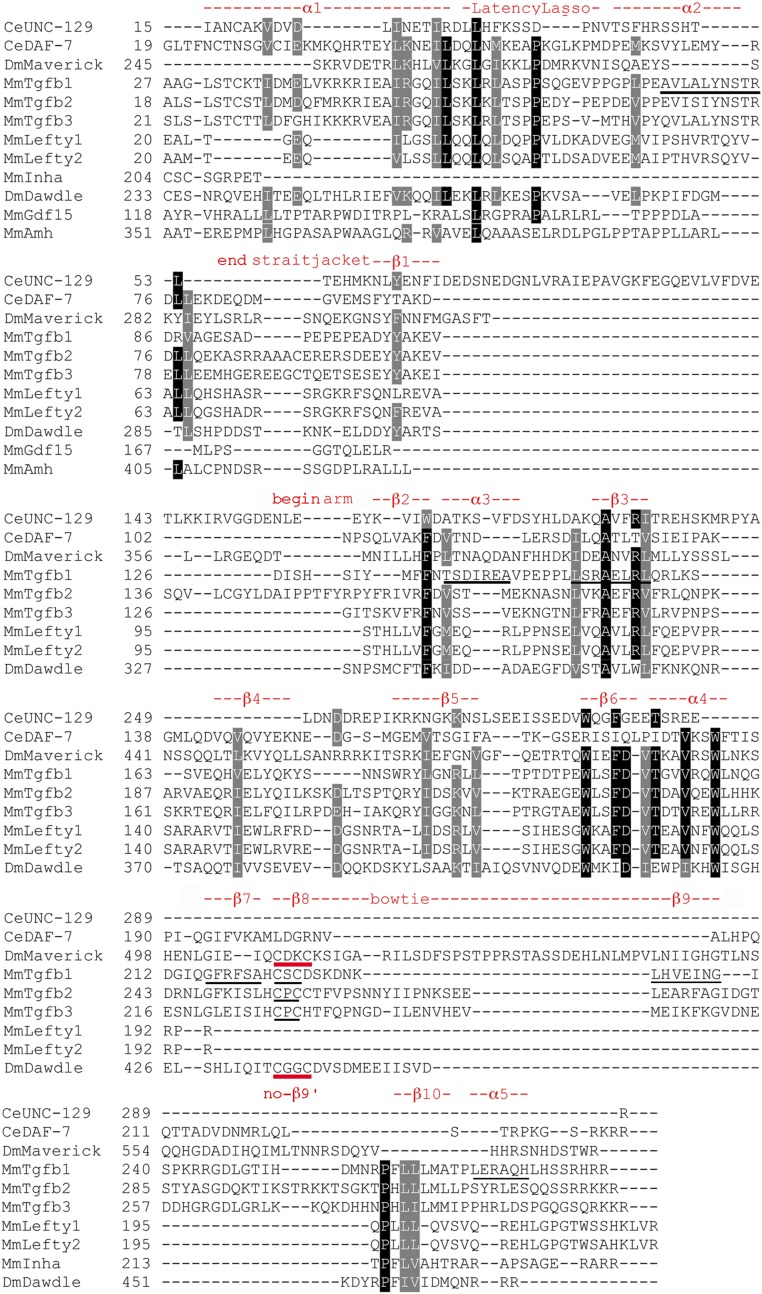
Focused TGF-β subfamily prodomain alignment indicating structural conservation. Sequences presented as in Figure 2. Numbering is accurate. The locations and naming of structural features are derived from TGF-β1 of Shi *et al.* (2011). The underlined α2 indicates a location distinct from α2 in Inhibin-βa. Underlined α3 and β3 indicate these features are in the reverse order *vs.* the Activin subfamily. The underlined features β7 and β9 of TGF-β1 do not appear conserved outside its two siblings. Unexpectedly, in β8 Maverick and Dawdle have a pair of cysteines (red underline) that align with those in TGF-β1–3 (black underline), although the spacing is not the same (CxxC *vs.* CxC). β9′ is not visible and may be unique to the BMP subfamily. The underlined α5 does not appear conserved.

Areas of structural conservation are evident in the annotated TGF-β alignments (Figure 4 and Figure S5). The locations and names of features derive from TGF-β1 (Shi *et al.* 2011). The four features that compose the straitjacket domain (α1, the Latency Lasso, α2, and β1) show less conservation than in the Activin subfamily. The first three features contain a set of nine prominent I/L/V residues with only one near universal. The proline in the Latency Lasso is only modestly conserved and β1 contains only a modestly conserved F/Y.

At the 5′ end of the arm domain, there are places within helices β2–6 and α4 that show more conservation than the Activin subfamily. β2 contains a near universal F/W, while β3 contains a near universal Alanine and I/L/V. β6 and α4 each have a near universal tryptophan and a near universal I/L/V. At the 3′ end of the arm, unexpectedly in β8 (part of the distinctive bowtie) Maverick and Dawdle have a pair of cysteines that align with those in TGF-β1–3, although the spacing is not the same (CxxC *vs.* CxC). β10 is modestly conserved. The distinct patterns of conservation in the Activin and TGF-β subfamilies support the idea that they are separate.

### Activin + TGF-β subfamily trees

**Figure 5:**
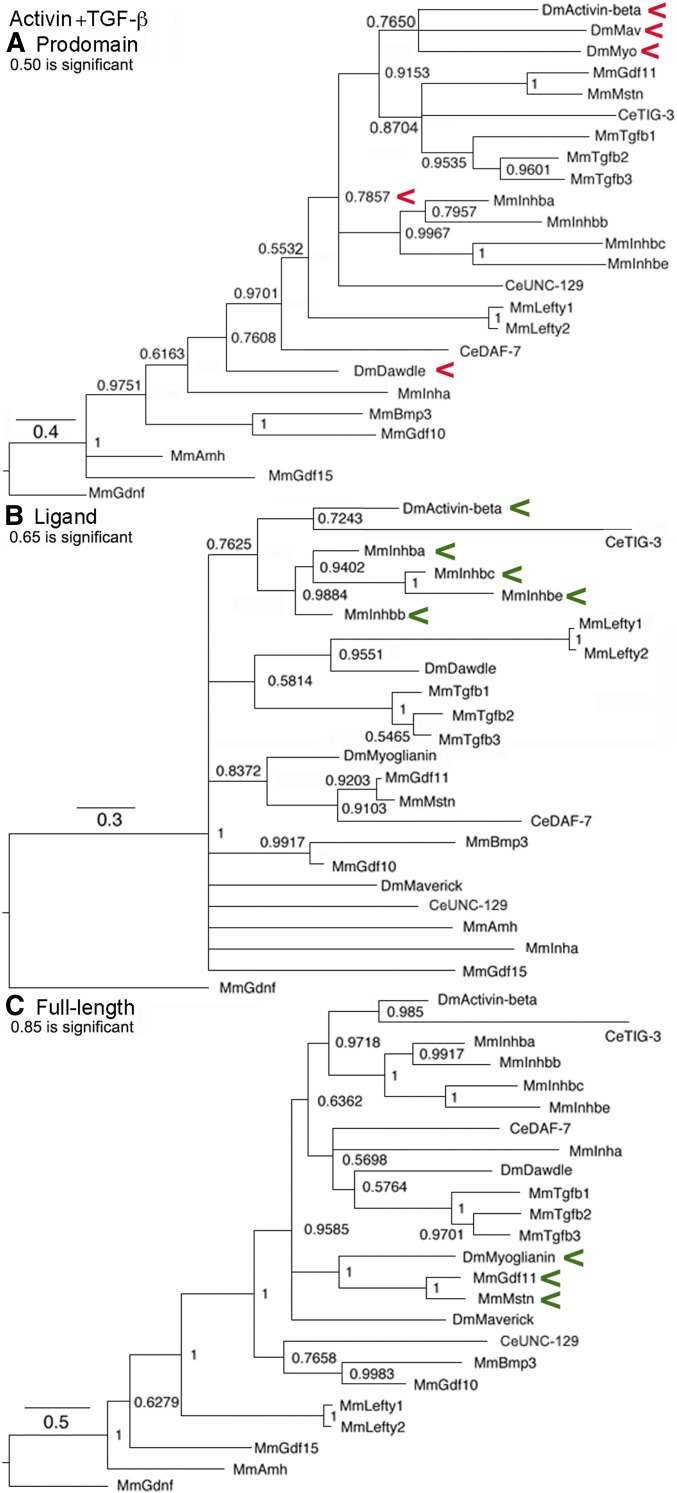
Activin + TGF-β subfamily trees. Bayesian trees of 23 sequences plus the outgroup are displayed as in Figure 1. Red arrowheads indicate a cluster that may reflect common regulation and green arrowheads a cluster that may reflect common function. (A) Prodomain nodes ∼0.50 are significant. The significant cluster of Activin, Maverick, and Myoglianin that is clustered with the four Inhibin-β proteins and Dawdle’s location near Inhibin-α were unexpected but consistent with cysteine conservation in the “LTBP-Association region” and β8. (B) Ligand nodes ∼0.65 are significant. The significant cluster of Activin and the four Inhibin-β proteins was expected. (C) Full-length nodes ∼0.85 are significant. The significant cluster of Myoglianin and Myostatin/GDF11 was expected.

The combined Activin + TGF-β subfamily (Figure 5) contains previously unsuspected relationships. The uniquely low threshold for node significance in the prodomain tree again demonstrates the distinct nature of these two subfamilies. The value needed for a node to attain statistical significance depends upon the number of informative sites in the underlying alignment. An informative site is one where an amino acid is present in virtually every family member with a different residue in at least two proteins. Thus, the large number of gaps needed to achieve prodomain alignment in the combined subfamily led to the smallest number of informative sites (*i.e.*, no other tree has a significance threshold as low as 0.50).

In the prodomain, one cross-subfamily cluster contains three of the four fly family members (Activin, Maverick, and Myoglianin). Further, as a group they are tightly tied in a second cross-subfamily cluster to TGF-β1–3 and Myostatin/GDF11. The group of four Inhibin-β proteins is the next closest cluster. Dawdle ends up as a solo next to Inhibin-α. The clustering of the three fly proteins with Dawdle as an outlier is reminiscent of the Inhibin-β group’s relationship with Inhibin-α, proteins that are known to heterodimerize. The analogy is that Dawdle can bind to Activin, Maverick, and Myoglianin and that these heterodimers have a distinct function (possibly inhibition) from the four homodimers (possibly activation).

On the other hand, in the ligand and full-length trees there are no cross-subfamily clusters, the Activin subfamily and TGF-β subfamily relationships are simply recreated. For example, Activin is with the four Inhibin-β proteins and Myoglianin is with Myostatin/GDF11. Overall for the Activin + TGF-β subfamily, similarity between the ligand tree and the full-length tree indicates that functional relationships of the ligand are driving its evolution.

### Activin + TGF-β subfamily prodomain structural conservation

**Figure 6:**
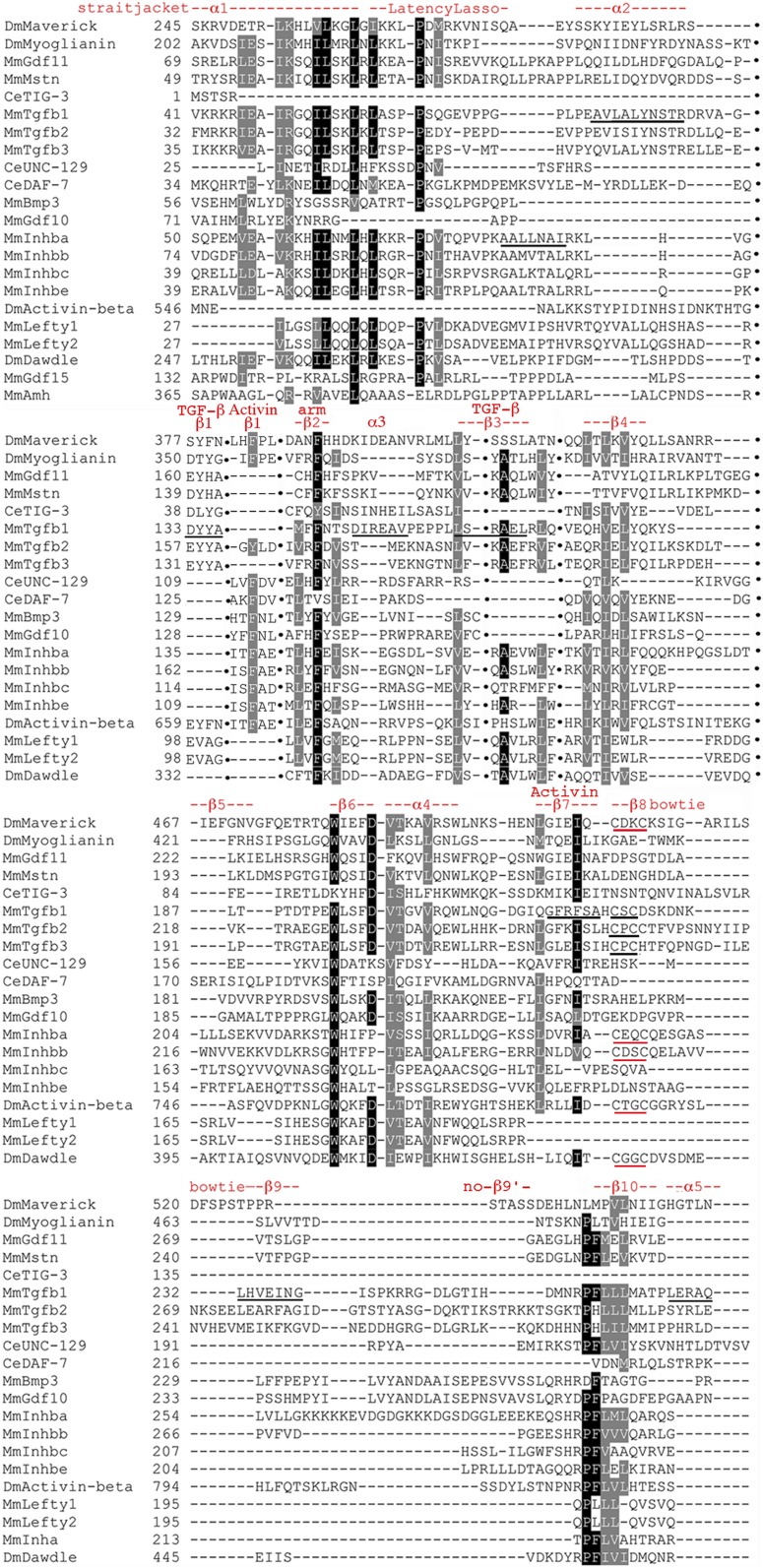
Focused Activin + TGF-β subfamily prodomain alignment indicating structural conservation. Sequences presented as in Figure 2 except gaps are omitted for brevity and indicated by columns of black dots. Numbering is only approximate. Locations and naming of structural features are from TGF-β1 (Shi *et al.* 2011). Underlined α2 sequences of TGF-β1 and Inhibin-βa indicate distinct locations. Underlined β1 in TGF-β1 shows it is in a distinct location in Inhibin-βa. Underline of α3 in TGF-β1 indicates absence of conservation. The notation TGF-β with β3 indicates this feature approximates the TGF-β1 pattern (underlined). The notation Activin with β7 indicates this feature approximates the Activin pattern (underlined) not the underlined TGF-β1 pattern. In β8 of the bowtie of TGF-β1–3, the conserved pair of cysteines (CxC) is underlined in black. In β8 Maverick and Dawdle are joined by Activin, Inhibin-βa, and Inhibin-βb, with a conserved pair of cysteines of distinct spacing (red underline; CxxC). Underlined β9, β9′, and α5 are not conserved.

The combined Activin + TGF-β subfamily alignment contains four features where the subfamilies differ, further supporting the conclusion that these are distinct (Figure 6 and Figure S6). The locations and names of features derive from TGF-β1 (Shi *et al.* 2011). The first two features of the straitjacket (α1 and Latency Lasso) are conserved. These contain seven I/L/V residues with one near universal. The proline in the Latency Lasso is also near universal. Each subfamily has a distinct location for α2 and for β1, although neither of the versions of α2 or β1 are conserved.

At the 5′ end of the arm, β2 contains a near universal phenylalanine. β3 is the third feature distinct in the Activin and TGF-β subfamilies. β3 shows conservation in the TGF-β pattern that pulls in several Activin subfamily members anchored by an alanine and an I/L/V. β4 has three near universal I/L/V residues. β6 has a near universal tryptophan and α4 a near universal I/L/V. At the 3′ end of the arm β7 is the fourth feature that is distinct, it shows conservation in the Activin pattern that pulls in several TGF-β subfamily members. β10 has a near universal proline, phenylalanine, and three I/L/V residues.

Again unexpectedly in β8, Maverick and Dawdle in the TGF-β subfamily are joined by Activin, Inhibin-βa, and Inhibin-βb in the Activin subfamily, with a pair of conserved cysteines having the same spacing (CxxC *vs.* CxC in TGF-β1–3). In all eight proteins the first cysteine is aligned. The fact that Activin, Dawdle, Maverick, Inhibin-βa, and Inhibin-βb have a pair of similarly spaced cysteines in β8 that mediates dimerization in TGF-β1, is consistent with the prodomain clusters that suggested cross-subfamily heterodimerization of Activin with Dawdle and Maverick. Importantly, the cysteines suggest a biochemical mechanism by which heterodimerization can be achieved.

### BMP subfamily trees

**Figure 7:**
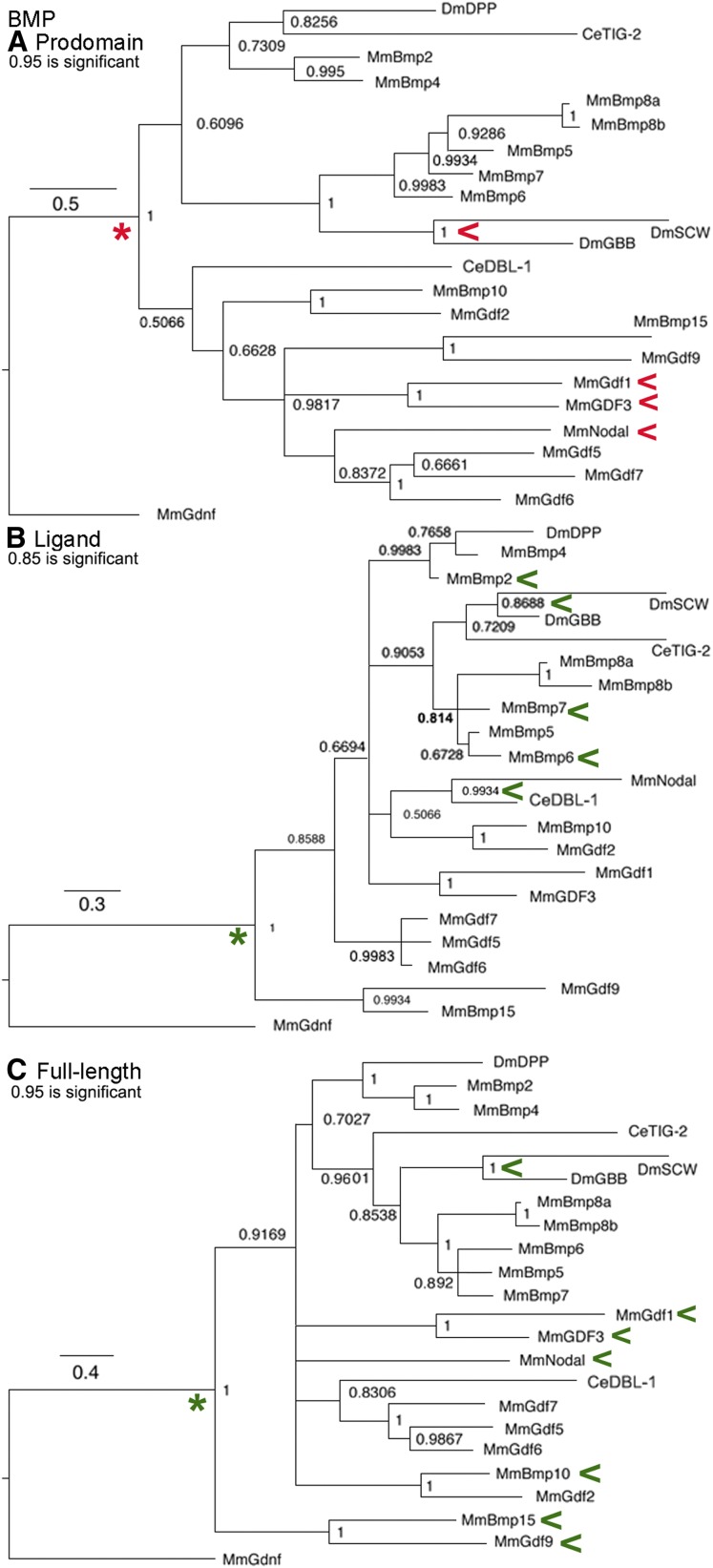
BMP subfamily trees. Bayesian trees of 22 sequences plus the outgroup are displayed as in Figure 1. Red arrowheads indicate a cluster that may reflect common regulation and green arrowheads a cluster that may reflect common function. (A) Prodomain nodes ∼0.95 are significant. The significant cluster of Gbb/Screw was unexpected. The cluster of heterodimerizing Nodal and GDF1/GDF3 was expected. Red asterisk indicates node leading to two symmetric secondary clusters. (B) Ligand nodes ∼0.85 are significant. The significant cluster of BMP2-8a, b with Gbb/Screw was expected and consistent with functional heterodimers of BMP2-BMP6 and BMP2-BMP7 that have been reported. The significant cluster of Nodal and DBL-1 was unexpected. Green asterisk indicates node leading to two asymmetric secondary clusters. (C) Full-length nodes ∼0.95 are significant. Clustering of GBB/Screw, BMP10-GDF9, BMP15-GDF9, and Nodal-GDF1/GDF3 are consistent with heterodimerization that has been reported. Green asterisk indicates node leading to two asymmetric secondary clusters.

This subfamily (Figure 7) is the largest of the three and is built upon the BMP2/BMP4 proteins that cluster together in all trees based on recent origin. An important finding is that in all trees Gbb and Screw are in a cluster, with the same statistical significance as BMP2/4 and other recent duplications. It appears that Screw resulted from divergence after the duplication of Gbb, uniquely in the lineage leading to *Drosophila*. For example in *Aedes* mosquitos, also a Dipteran, there is no Screw but instead two copies of Gbb (Leiber and Luckhart 2004). Both Gbb and Screw form heterodimers with Dpp during development (*e.g.*, Shimmi *et al.* 2005).

A corollary of Gbb/Screw clustering is that the within subfamily clustering of Gbb with mouse BMP5–8a,b proteins is now extended to Screw as is shown in all trees. A second corollary is that each of the BMP5–8a,b proteins may have the ability to heterodimerize with BMP2/4 yielding as many as 10 possible combinations. To date, only two of these heterodimer pairs have been reported: BMP2/BMP7 in zebrafish dorsal-ventral axis formation (Little and Mullins 2009), and BMP2/BMP6 in mammalian osteogenesis (Loozen *et al.* 2018). Outside this group, heterodimers of mammalian BMP10/GDF9 regulate vascular remodeling (Tillet *et al.* 2018).

Nodal has distinct but not significant clusters in the prodomain with GDF5-7 that link significantly to two mammalian pairs BMP15/GDF9 and GDF1/GDF3 and the triplet GDF5/GDF6/GDF7. Heterodimers of BMP15/GDF9 were seen *in vitro* in rat follicle cell assays that signaled through a cross-subfamily complex of the BMP Type II receptor BMPR2 and the Activin Type I receptor ACVR1B (McIntosh *et al.* 2008). It was recently reported that Nodal heterodimers with GDF1 are required for mesoderm induction in zebrafish (Montague and Schier 2017). Based on extensive coexpression of GDF1 and its duplicated partner GDF3, the authors propose Nodal/GDF3 heterodimers are functional in other developmental contexts.

Interestingly, the overall topology of the BMP prodomain tree is different from the others. In the ligand and full-length trees there are two asymmetric secondary clusters, everyone *vs.* BMP15/GDF9, suggesting one predominant function. The prodomain tree has two symmetric secondary clusters. One cluster is BMP proteins related to Dpp, Gbb, and Screw, while the other is GDF proteins plus Nodal. Note that BMP10 and BMP15 (also known as GDF9b) have names that do not fit their association with GDF proteins. The prodomain tree suggests that perhaps each major cluster has a distinct mode of regulation.

In the ligand tree Nodal is significantly paired with nematode DBL-1 and then loosely with the mammalian pair BMP10/GDF2. The tight association between mouse Nodal and DBL-1 in the absence of any fly protein is curious. Nodal’s distinct ligand and prodomain associations lead it to be a loner in the full-length tree. Otherwise, the full-length BMP tree largely shows previously established clusters. Overall for the BMP subfamily, similarity between the ligand and full-length trees indicate that functional relationships of the ligand are driving its evolution.

### BMP subfamily prodomain structural conservation

**Figure 8:**
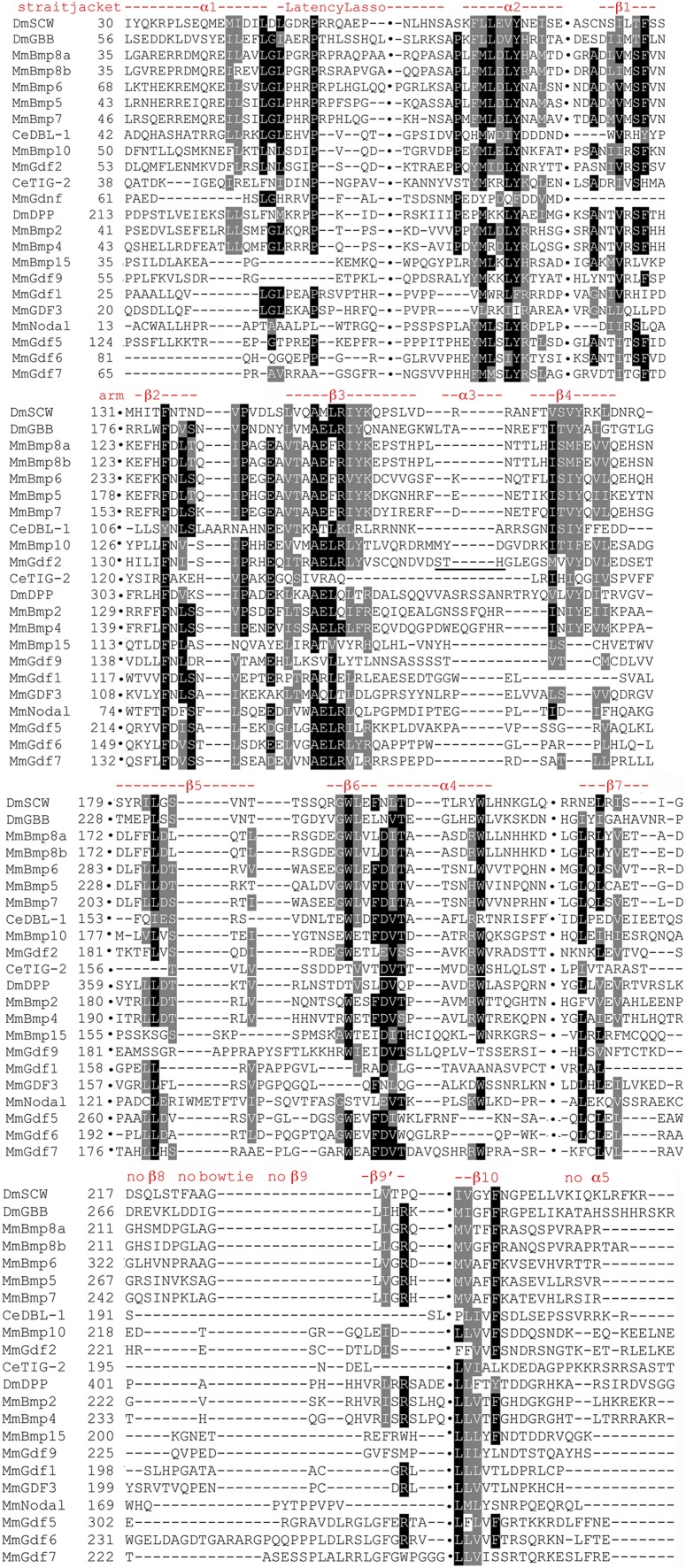
Focused BMP subfamily prodomain alignment indicating structural conservation. Sequences presented as in Figure 6 with gaps indicated by columns of black dots and approximate numbering. The locations and naming of structural features are from BMP9 (Mi *et al.* 2015), shown here by its synonym GDF2. The absence of α3 is indicated by the underlining in GDF2. The features β7 and β9′ are conserved in this subfamily, while β8, β9 (the distinctive bowtie), and α5 of TGF-β1 are not.

The BMP subfamily appears more homogeneous than the Activin or TGF-β subfamilies in the annotated alignments (Figure 8 and Figure S7). Homogeneity is evident in a larger number of conserved residues and a greater frequency of identical residues. The locations and names of features in this subfamily derive from BMP9 (Shi *et al.* 2011; included here as GDF2). All of the features of the straitjacket (α1, Latency Lasso, α2, and β1) display strong homogeneity. α1 and the Latency Lasso contain 10 conserved I/L/V residues with three that are near universal. There is a pair of near universal F/Y residues in α2. An F/Y and the adjacent S/T in β1 are moderately conserved.

At the 5′ end of the arm strong conservation is visible. β2 contains near universal F/Y and I/L/V residues. Conservation is present between β2 and β3 with a near universal I/L/V and a modestly conserved proline. β3 has a stretch of seven consecutive conserved residues, a degree of continuous conservation not seen previously. This stretch includes two near universal I/L/V residues, near universal R/K, alanine and glutamic acid residues, as well as modestly conserved R/K and F/Y. β4 also has a stretch of seven residues highly conserved in 66% of family members: four I/L/V residues, an S/T, and an F/Y. β5 is only moderately conserved with one near universal I/L/V. The β6 to α4 region has a highly conserved stretch of seven consecutive residues including near universal tryptophan, phenylalanine, aspartic acid, and S/T. There is a near universal tryptophan at the distal end of α4.

At the 3′ end of the arm, β7 has three moderately conserved I/L/V residues. β8 and β9 (the distinctive bowtie of TGF-β1) are not conserved. β9′ is a unique BMP feature containing a moderately conserved arginine and an I/L/V. β10 contains two near universal I/L/V residues and a modestly conserved phenylalanine. Overall, in the BMP subfamily the 5′ ends are more highly conserved than the 3′.

### All family members trees

**Figure 9:**
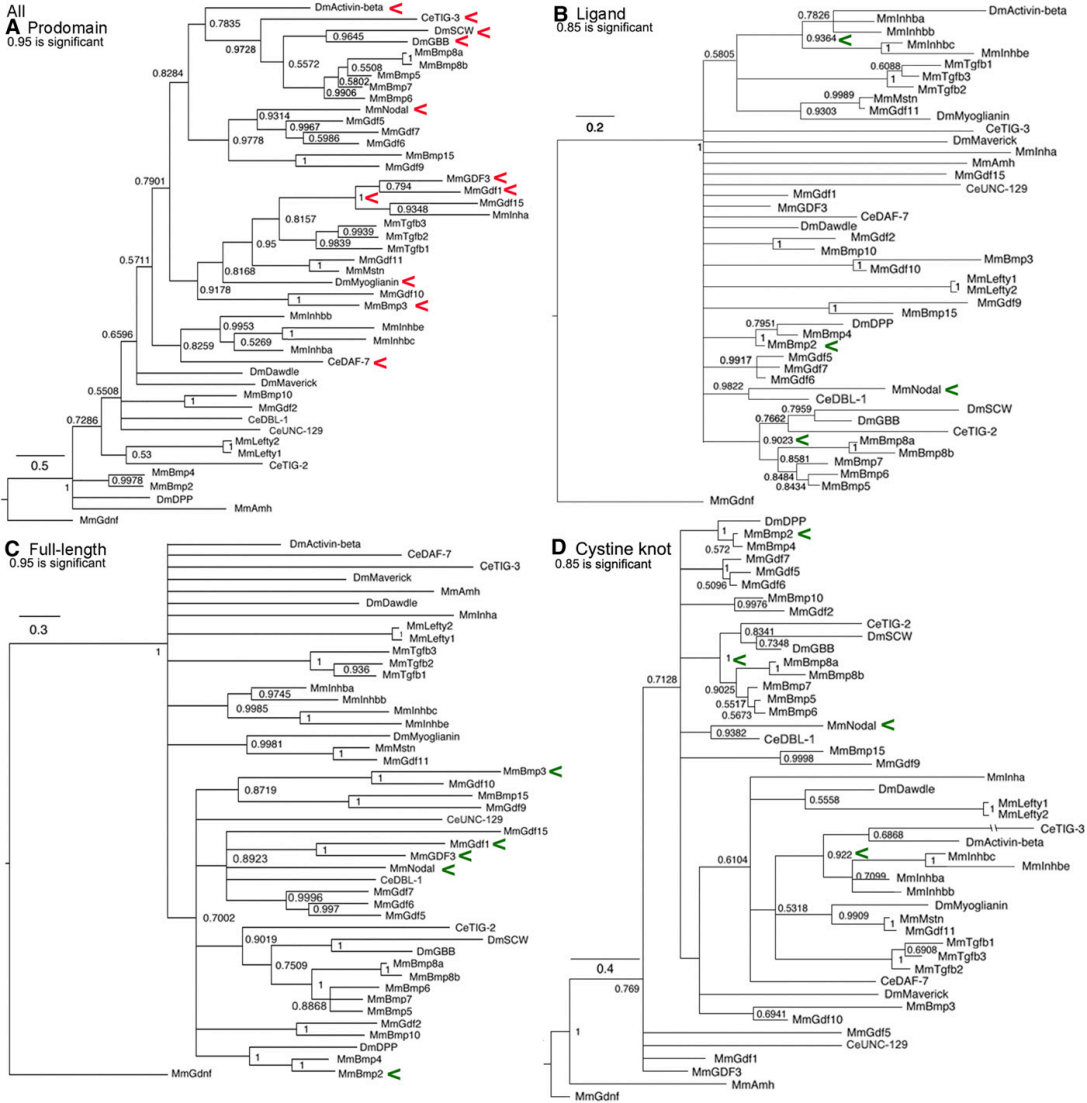
All family members trees. Bayesian trees of all 45 sequences plus the outgroup are displayed as in Figure 1. Red arrowheads indicate a cluster that may reflect common regulation and green arrowheads a cluster that may reflect common function. (A) Prodomain nodes ∼0.95 are significant. The not quite significant cross-subfamily cluster of Activin, TIG-3, Gbb, and Screw with Nodal was unexpected but three are known to heterodimerize and two have conserved cysteines. The absolute cluster of GDF3/GDF1 with GDF15/Inhibin-α and this group’s not quite significant cluster with Myoglianin was unexpected. The not quite significant cluster of DAF-7 with the four Inhibin-β proteins was unexpected but is consistent with “LTBP-Association region” cysteine conservation. The cluster of BMP3/GDF10 with Myoglianin was unexpected. (B) Ligand nodes ∼0.85 are significant. Several significant clusters are expected such as the four Inhibin-β proteins and Dpp/BMP2/BMP4. The significant cluster of Nodal and DBL-1 was unexpected. (C) Full-length nodes ∼0.95 are significant. The not quite significant clustering of BMP3 and GDF1 with all of the BMP subfamily proteins was unexpected. The not quite significant cluster between Nodal and GDF1/GDF3 was expected. (D) Cystine knot nodes ∼0.85 are significant. Several significant clusters are expected, such as Activin with four Inhibin-β proteins, Dpp/BMP2/BMP4 and Gbb/Screw/BMP5–8a,b. A significant cluster of Nodal and DBL-1 was unexpected.

Trees of the whole family (Figure 9) including a cystine knot tree to compare to the biochemically defined ligand tree are shown. A comparison of the latter two trees demonstrates the loss of resolution resulting from adding the degenerate spacer to the cystine knot alignment.

In the prodomain trees, there are six cross-subfamily clusters. One has Activin in a cluster with Gbb and Screw that heterodimerize with Dpp, although the node is just short of significant (0.78 *vs.* 0.95 for significance). What this modest shortfall means is that this is the best, but not the only possible placement of Activin in the tree. This unexpected cluster forms a second (although still not significant, 0.83 *vs.* 0.95), larger cross-subfamily cluster with Nodal and its closest GDF relatives in the BMP subfamily. While not conclusive, the first cluster suggests the hypothesis that the prodomains of Activin, Gbb, and Screw are similar as a result of the shared ability to heterodimerize with Dpp. The inclusion of BMP5–8a,b and Nodal with its closest GDF relatives in the larger second cluster suggests a level of similarity not found in the wider family. The most parsimonious hypothesis for this similarity is intracluster heterodimerization, implying numerous functional heterodimers yet to be identified.

A third cross-subfamily cluster in the prodomain is strongly although not significantly connected to the one above (0.79 *vs.* 0.95 for significance). This group contains the BMP subfamily members GDF3/GDF1 joined with absolute confidence (node = 1.0) to the TGF-β subfamily members Inhibin-α/GDF15. Together these form a fourth cross-subfamily cluster with significant, or just below significant, nodes to the TGF-β proteins and five Activin subfamily members (Myoglianin, Myostatin/GDF11, and BMP3/GDF10). This cluster suggests that these prodomains are similar to Inhibin-α as result of the ability to heterodimerize. This prediction is supported by Nodal heterodimers with GDF1/GDF3 (Montague and Schier 2017). The prodomain clustering of Nodal partners GDF1/GDF3 with the TGF-β subfamily members Inhibin-α/GDF15 could explain the ability of BMP subfamily Nodal/GDF1 heterodimers to signal via the TGF-β subfamily receptor ActRIIA.

A fifth cross-subfamily cluster in the prodomain is DAF-7 (TGF-β subfamily) with heterodimerizing Inhibin-β proteins. A sixth cross-subfamily cluster is TIG-3 (Activin subfamily) with heterodimerizing Gbb/Screw. These clusters suggest possible heterodimerization for these nematode proteins, the only prediction of this type for this species.

For the biochemically defined ligand tree, aside from recent duplications such as Lefty1/2, only three small secondary and three small tertiary clusters are seen. These are all composed of the most conserved proteins such as Activin/Inhibin-β group, Dpp/BMP2/4, and Gbb/Screw/BMP5–8a,b. Ten proteins are solos and Nodal is again paired with DBL-1.

By comparison, the cystine knot tree shows better resolution with only five proteins as solos. Unsurprisingly, secondary clusters of the same highly conserved proteins are visible. Surprisingly, Nodal is again paired with DBL-1. This is because the spacer of Nodal and DBL-1 shows unexpected conservation. The 11 amino acids closest to the first cysteine in DBL-1 contain seven of the 10 amino acids in the Nodal spacer (Table S3). This likely explains their consistent pairing in the biochemically defined ligand and cystine knot trees.

In the full-length tree, the Activin subfamily members BMP3/GDF10 are not quite significantly associated with the expanded group of BMP subfamily members. However, they show the same level of association with the TGF-β subfamily in the prodomain and are solos in the cystine knot and ligand trees. This combination of placements suggests that for BMP3/GDF10 their regulation and function share features of distinct subfamilies that will need to be identified by experiment. The pair of BMP subfamily members GDF1/GDF3 that heterodimerize with Nodal also have features of multiple subfamilies. They are significantly clustered to the TGF-β subfamily members Inhibin-α/GDF15 in the prodomain tree, solos in the cystine knot and ligand trees, and not quite significantly associated with the expanded group of BMP subfamily members in the full-length tree. As noted above, the association of GDF1/GDF3 prodomains with Inhibin-α/GDF15 may explain why Nodal heterodimers with GDF1/GDF3 can signal through ActRIIA.

The placement of Nodal and DBL-1 as solos in the full-length tree is distinct from the ligand and cystine knot trees where they are a significant pair and the prodomain tree where they are essentially unlinked. These two proteins likely have homologous receptors but distinct regulation and it is a specific combination of regulation and function not found in any fly protein.

In the full-length tree, four fly proteins Activin and Myoglianin in the Activin subfamily, plus Dawdle and Maverick in the TGF-β subfamily, are solos (with Myoglianin sticking to its mammalian partners). This contrasts with the prodomain tree where Activin is in a weak BMP cluster and Myoglianin in a weak TGF-β cluster. Alternatively, these two are weakly linked to an Activin + TGF-β cluster in the cystine knot and ligand trees. Their distinct placement in these trees suggests the four fly proteins have mechanisms of regulation yet to be identified. Overall, the many solos in the full-length trees result from dissimilarity between the cystine knot, ligand, and prodomain trees. This indicates that functional and regulatory relationships are equally driving the evolution of the TGF-β family.

### All family members prodomain structural conservation

**Figure 10:**
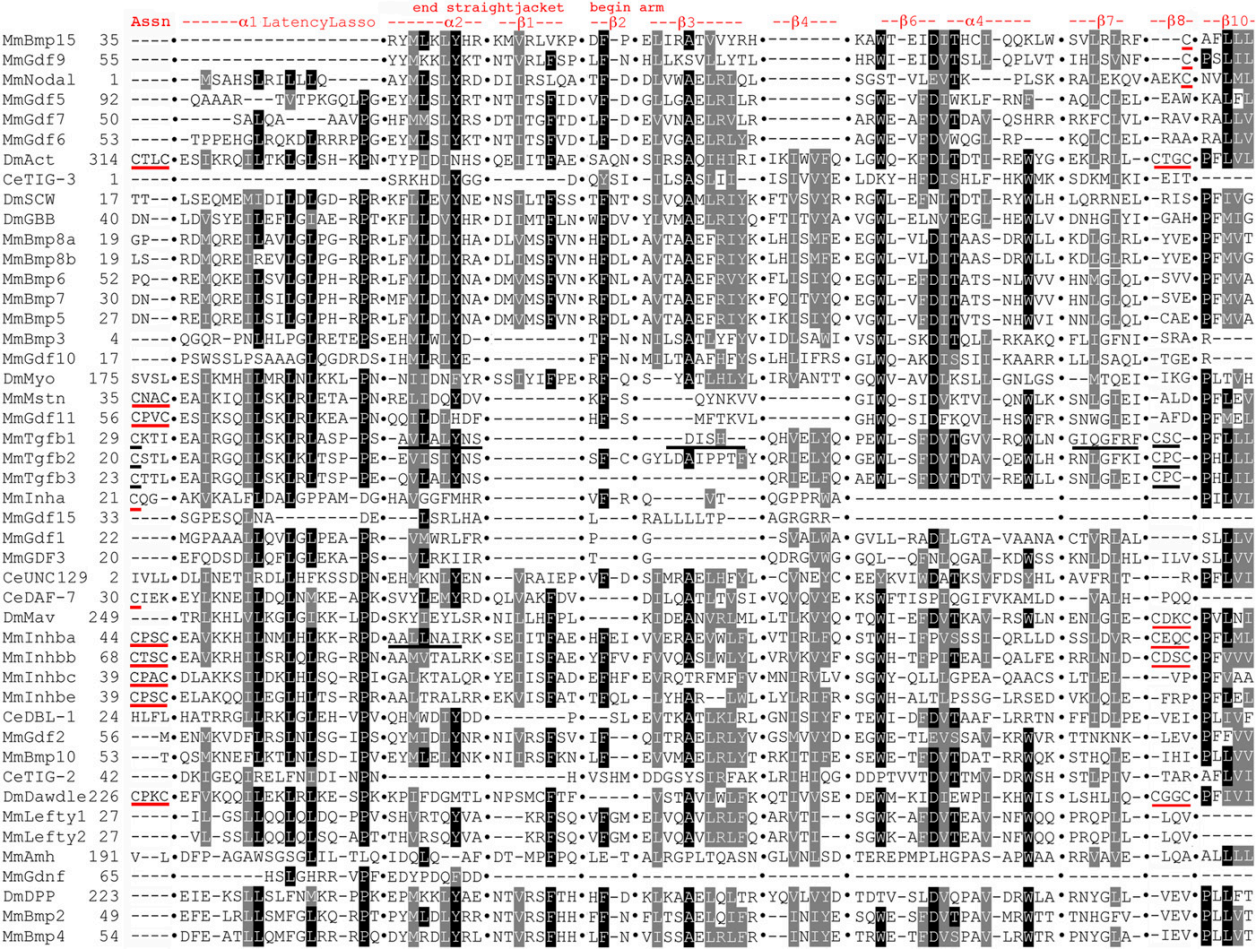
Focused All family members prodomain alignment indicating structural conservation. Sequences presented as in Figure 6 with gaps indicated by columns of black dots and approximate numbering. Only highly conserved features are shown for brevity. The locations and naming of structural features are derived from TGF-β1 and BMP9 as noted above with the latter shown here by its synonym GDF2. In the “LTBP-Association region”, the cysteines in TGF-β1–3 that form a disulfide bond with LTBP are underlined in black. The unexpectedly conserved pairs of cysteines in Activin, Dawdle, the four Inhibin-βs, and Myostatin/GDF11 are underlined in red. Note that the first cysteine of each pair aligns with the cysteine of TGF-β1–3. The conserved cysteines of Inhibin-α and DAF-7 are also underlined in red. The two underlines of α2 show that TGF-β1 and Inhibin-βa have been pulled into alignment by the homogeneity of the Activin + BMP subfamilies. The Activin + BMP version of β1 is conserved but the TGF-β1 version is not. The underlining of TGF-β1 in β3 and β7 shows that the Activin + BMP version is conserved, while the TGF-1β version is not, although TGF-β subfamily members have been pulled into alignment by the homogeneity of the Activin + BMP subfamilies. In β8, the pair of cysteines in TGF-β1 and its siblings (CxC) are underlined in black. The unexpectedly conserved pairs of cysteines in Activin, Maverick, Dawdle, Inhibin-βa, and Inhibin-βb (CxxC) are underlined in red. Note that the first cysteine of the CxxC aligns with the first cysteine of the CxC. The unexpectedly conserved single cysteines of Nodal, BMP15/GDF9, are also underlined in red. Note that these single cysteines align with the second cysteine of the CxxC.

The conservation pattern in the subfamilies is reiterated in the annotated All family members alignment (Figure 10 and Figure S8). In the straitjacket α1, the Latency Lasso and α2 display strong conservation. These contain nine conserved I/L/V residues with the second, third and fourth nearly universal and nearly always leucine. There is a near universal proline in the Latency Lasso and a tyrosine in α2.

The α2 region of TGF-β1 contains three conserved I/L/V residues (one universal) and a tyrosine. These are not conserved in the TGF−β subfamily or the Activin + TGF-β subfamily. Alternatively, two to four of these amino acids (VL_LY) are nearly universal in the BMP subfamily. BMP conservation has driven the alignment of All family members to identify these amino acids in α2 of the other subfamilies. In other words, the All family members alignment erases the distinction in the location of α2 in the Activin and TGF-β subfamilies. In β1, one I/L/V and a phenylalanine present in the Activin + BMP subfamilies are absent in the TGF-β subfamily, maintaining the prior distinction in β1 location and conservation.

The 5′ end of the arm is not well conserved. β2 contains only a modestly conserved phenylalanine. β3 is conserved in an Activin + BMP pattern that draws in several TGF-β subfamily members, although not TGF-β1. The previously identified stretch of seven conserved residues in β3 is reduced to six having lost the glutamic acid. β4 conservation is also reduced, with its stretch of seven residues now only three: two I/L/V residues and an F/Y. β4 is absent in Nodal and seven other BMP subfamily members. The middle region of the arm, β6 to α4 is the best conserved part, yet it too shows a reduction. The highly conserved stretch of seven consecutive residues is at most four and often just two or three. The near universal tryptophan that was previously the first of the seven conserved residues is now separated by two or more nonconserved amino acids from the core of two to four residues (phenylalanine, aspartic acid, I/L/V, and threonine). The aspartic acid is well conserved. The near universal tryptophan at the distal end of α4 is still present.

At the 3′ end of the arm conservation is also limited. β7, like β3, is conserved in an Activin + BMP pattern that draws in several TGF-β subfamily members, although not TGF-β1. β7 retains two moderately conserved I/L/V residues of three previously. β10 is the best conserved feature in the region, perhaps anchoring the 3′ end of the protein. There are two near universal and a modestly conserved I/L/V plus a modestly conserved proline. Overall, the 5′ end of the straitjacket (α1, Latency Lasso, and α2) plus the central β6 to α4 and 3′ end of the arm (β10) are the six best conserved features of the 17 prodomain features in the TGF-β family.

Within β8 of the bowtie of TGF-β1 is the previously noted conservation of a pair of cysteines in eight Activin + TGF-β subfamily members (either CxxC or CxC with the position of the first cysteine aligned). Unexpectedly, three proteins in the BMP subfamily (BMP15/GDF9 and Nodal) have a single cysteine in β8 that aligns with the second cysteine of the CxxC (underlined in Figure 10 and Figure S8, page 4). All members of the BMP trio with a conserved cysteine are known to heterodimerize: BMP15/GDF9 with each other (McIntosh *et al.* 2008) and Nodal with GDF1 (Montague and Schier 2017). Also, these three form a significant secondary cluster in the BMP prodomain tree. The confluence for these three proteins of conserved cysteines in the alignments, a significant prodomain cluster and experimentally demonstrated heterodimerization serves as a proof of principle for our approach and heterodimerization predictions for other family members.

The discovery of cysteine conservation in β8 in all three subfamilies reminded us that TGF-β1–3 prodomains are often covalently linked via a cysteine bridge to LTBPs (Rifkin *et al.* 2018). We easily identified the conserved solo cysteines in the “LTBP-Association region” near the amino terminus of TGF-β1–3 (underlined in Figure 10 and Figure S8, page 1). In our alignment, the “LTBP-Association region” contains a conserved pair of cysteines in Activin, the four Inhibin-βs, Dawdle, and the duplicated pair Myostatin/GDF11. The first cysteine of the pair in each of these eight proteins from the Activin and TGF-β subfamilies is aligned with the cysteine of TGF-β1–3. Further, a single cysteine is present in Inhibin-α and DAF-7 that also aligns with the cysteine of TGF-β1–3. A total of 10 proteins in the Activin + TGF-β subfamilies appear capable of covalent linkages via the “LTBP-Association region”.

Taken together, a total of 14 proteins from all three subfamilies display cysteine conservation in regions associated with dimerization (β8) or protein–protein interactions (“LTBP-Association region”). Many of these cysteine containing proteins are predicted by prodomain clustering to heterodimerize such as Activin and Dawdle. Interestingly, both of these proteins have conserved cysteines in β8 and the “LTBP-Association region”, suggesting the possibility of multiple heterodimerization partners.

## Discussion

### Prodomain structure conservation

Across the prodomain alignments, distinctions in the conservation of structural features between the subfamilies are seen in both the straitjacket and arm domains. In the straitjacket there are discrepancies between the Activin and TGF-β subfamilies in the locations of α2 and β1. At the boundary of the straitjacket and arm, a third distinction between the Activin and TGF-β subfamilies is the order of α3 and β3 (Activin has β3 first and TGF-β has α3 first). In the arm there are three additional differences. β3 and β7 show dissimilarities between the Activin + BMP subfamilies and the TGF-β subfamily. β9′ is distinct between the BMP and Activin + TGF-β subfamilies. If any functional differences are engendered by these structural distinctions, then they are unknown at this time.

The discovery of a conserved pair of cysteines in β8 in five Activin + TGF-β subfamily members and a single conserved cysteine in three BMP subfamily members is exciting. From an evolutionary perspective two points can be made. First, the presence of conserved cysteines in β8 in all subfamilies suggests that prodomain participation in protein–protein interactions is an ancient mechanism. The closed-ring conformation of TGF-β1 employing a bowtie in β8/β9 to mediate dimerization is a recent innovation built upon this foundation. Second, the nonuniversality of β8 cysteine conservation suggests significant within-subfamily structural variation between cysteine-bearing and noncysteine-bearing proteins. Structures are known only in the Activin subfamily for Inhibin-βa that has these cysteines and in the BMP subfamily only for BMP9 that does not have them. Analysis of additional family members may reveal additional conformations.

Similar excitement is generated by the discovery of a conserved pair of cysteines in the “LTBP-Association region” in eight Activin + TGF-β subfamily members and a single conserved cysteine in two additional members of the TGF-β subfamily. Interestingly, no BMP subfamily members have conserved cysteines here. One caveat is that prodomain length upstream of the straitjacket in the BMP subfamily is highly variable (from one residue in Nodal to 223 in Dpp). There might be functional cysteines that are not close enough to the Activin + TGF-β subfamily cysteines to be captured in the alignment.

The presence of conserved cysteines in all Inhibin-βs and Inhibin-α suggests the obvious hypothesis that they participate in cross-subfamily heterodimerization. A structural analysis of these heterodimers should be fruitful A logical extension of this hypothesis is the heterodimerization of Activin and Dawdle. For the latter, beyond the “LTBP-Association region” this hypothesis is supported by three pieces of evidence: LTBP does not exist in flies and cannot utilize this cysteine, Activin-Dawdle heterodimerization was predicted in numerous prodomain trees, and these proteins also share β8 cysteine conservation. When four lines of computational evidence converge, confidence in the hypothesis is very high.

### Predicted heterodimerization

Although previously we noted *a priori* that we considered prodomain clustering as evidence of heterodimerization and thus common regulation, our review of existing literature in light of the identified clusters suggests that heterodimerization can also influence function. There are a number of examples where heterodimers function distinctly from constituent homodimers, with the Inhibins being the most prominent. Here we consider prodomain clustering, whether within or between subfamilies, to suggest new hypotheses for common regulation and/or distinct function via heterodimerization.

One hypothesis for a distinct function of heterodimers suggests a mechanism for TGF-β ligands’ ability to stimulate their receptors to activate non-Smad pathways such as the MAP-kinase, Rho-like GTPase, and PI3-kinase/AKT pathways. Currently, cell type–specific accessory proteins such as Par6 are considered responsible for a receptor’s choice of signal transduction pathway [reviewed in Zhang (2009)]. Prodomain clustering suggests that the choice may also be influenced by ligand heterodimers, a possibility that has not been previously considered.

In addition to non-Smad pathway activation, functional discrepancies for subfamily dedicated receptors and receptor-associated Smads have been noted. In flies, the Activin/TGF-β dedicated Type I receptor Baboon can signal through the BMP Smad protein Mad (Gesualdi and Haerry 2007; Peterson *et al.* 2012; Peterson and O’Connor 2013). Also in flies, BMP ligands can bind to the Activin/TGF-β Type II receptor Wit (Lee-Hoeflich *et al.* 2005). In mammals, Inhibin-β homodimers can bind to the Type II receptor BMPRII (Rejon *et al.* 2013). The mechanisms underlying these cross-subfamily interactions remain largely unknown. One could speculate that they are influenced by heterodimers resulting from cross-subfamily prodomain similarity. Nodal heterodimerization may serve as an example as its partners GDF1/GDF3 have prodomains that cluster with Inhibin-α/GDF15 suggesting a mechanism for Nodal signaling through ActRIIA.

Overall, we identified six cross-subfamily and two within-subfamily clusters that suggest previously unsuspected heterodimers. In every cross-subfamily cluster at least one protein with an unexpectedly conserved cysteine is involved. For example Activin, that has both association region and β8 cysteines participates in multiple cross-subfamily clusters.

### Predicted fly heterodimers for activin

To date, there is no evidence in the literature that consideration has been given to the possibility that Activin functions as a heterodimer. This is surprising since Activin owes its name to its closest relatives, the heterodimerizing Inhibin-β proteins (Inhibin-βa synonym is Activin-A). The prodomain trees contain clusters that suggest multiple heterodimer partners for Activin.

First is the prodomain cross-subfamily cluster of Activin, Myoglianin, and Maverick in the Activin + TGF-β tree. This cluster has strong statistical support. In the same tree Dawdle is adjacent to Inhibin-α, the heterodimerization partner of Activin’s Inhibin-β relatives. The Dawdle and Inhibin-α relationship is statistically significant. This pair of clusters suggests that Activin as well as Myoglianin and Maverick can form heterodimers with Dawdle. The heterodimerization predicted by this cross-subfamily cluster is strongly supported by structural conservation: conserved cysteines in β8 of Activin, Maverick, and Dawdle; and conserved cysteines in the “LTBP-Association region” of Activin and Dawdle.

Second is the prodomain cross-subfamily cluster in the All family members tree of Activin with Gbb and Screw, two proteins known to heterodimerize with Dpp. While not quite at statistical significance, the explanatory power of this cluster is welcome. Recently Dpp from imaginal tissues was shown to circulate in the hemolymph to reach the prothoracic gland where it influenced steroid hormone biosynthesis via its typical pathway (Setiawan *et al.* 2018). Circulation is an unprecedented role for Dpp, but a well-established one for Activin (*e.g.*, Gibbens *et al.* 2011). This cross-subfamily cluster suggests that circulating Dpp is actually a heterodimer with Activin and that Dpp targets the prothoracic gland via an Activin-based mechanism. This adds to the suggestion that Activin does many of its jobs as a heterodimer.

### Predicted heterodimers for nodal and nematode proteins with potential convergence

For Nodal, our data may explain one of its puzzles but also reveals a new one. A cross-subfamily cluster in the All family members prodomain tree may explain Nodal’s ability to signal through the TGF-β receptor ActRIIA. This is a cross-subfamily cluster that links the BMP subfamily members GDF3/GDF1 with absolute confidence to the TGF-β subfamily members Inhibin-α/GDF15. These four are in a second larger cross-subfamily cluster with the prototype TGF-β proteins and five Activin subfamily members. All the proteins in the larger cluster, except GDF3/GDF1, signal through TGF-β receptors such as ActRIA and ActRIIA (ten Dijke *et al.* 1994). GDF1/GDF3 may be included in this cluster because, like the others, their prodomain provides the ability to signal through TGF-β receptors. Nodal heterodimers with GDF1/GDF3 that signal via TGF-β receptors could explain Nodal signaling through ActRIIA.

The new puzzle is embodied in the statistically supported cluster of the BMP subfamily members Nodal and DBL-1 in the All family members ligand and cystine knot tree but not in any prodomain or full-length tree. Contributing to this cluster in our ligand and cystine knot trees is the unexpected conservation of their spacers. Another level of incongruity for this cluster is the absence of a fly protein. Two logical explanations for this cluster are that Nodal/DBL-1 are identical by descent and the fly counterpart has been lost, or convergent evolution of Nodal/DBL-1 based on shared function.

Evidence supporting convergence is the fact that conservation of the Nodal/DBL-1 spacer region (70% similarity, 40% identity) exceeds that of documented homologs Dpp/BMP2 (50% similarity, 15% identity) and Dpp/BMP4 (21% similarity; 0% identity), notwithstanding the 30% longer divergence time between nematodes and mammals than between flies and mammals (Hedges *et al.* 2004). We hesitate to speculate on the basis for convergence as it could be due to receptors or coreceptors either known or yet to be identified, or a completely unanticipated feature of their signaling pathways.

Additional clarity regarding DBL-1 comes from the BMP subfamily trees. In every tree, the unstudied TIG-2 is substantially closer to Dpp/BMP2/BMP4 than DBL-1. Thus DBL-1 is not the BMP2/BMP4 homolog, even though this outdated view is enshrined in GenBank (#AAC27729).

A new hypothesis for DAF-7 is provided by two sets of cross-subfamily clusters in the All family members prodomain tree: DAF-7 with the Activin subfamily heterodimerizing Inhibin-β group and TIG-3 with heterodimerizing Gbb/Screw. Together these two clusters suggest the possibility of cross-subfamily heterodimerization between DAF-7 and the unstudied TIG-3.

In summary, the prodomain alignments revealed that six structural features are well conserved: three in the straitjacket and three in the arm. Alignments also revealed unexpected cysteine conservation in the “LTBP-Association region” upstream of the straitjacket and in β8 of the bowtie in 14 proteins from all three subfamilies. In prodomain trees, eight clusters across all three subfamilies were present that were not seen in the ligand or full-length trees, suggesting prodomain-mediated cross-subfamily heterodimerization. Consistency between cysteine conservation and prodomain clustering provides support for heterodimerization predictions. Overall, our analysis suggests that cross-subfamily interactions are more common than currently appreciated, and our predictions generate numerous testable hypotheses about TGF-β function and evolution.
